# Curcumin Disrupts a Positive Feedback Loop between ADMSCs and Cancer Cells in the Breast Tumor Microenvironment via the CXCL12/CXCR4 Axis

**DOI:** 10.3390/pharmaceutics15112627

**Published:** 2023-11-15

**Authors:** Bo-Young Jang, Min Kyoung Shin, Dong-Hee Han, Jung-Suk Sung

**Affiliations:** Department of Life Science, Dongguk University-Seoul, Goyang 10326, Republic of Korea; by200015@naver.com (B.-Y.J.); samantha1994@naver.com (M.K.S.); hdh0715@naver.com (D.-H.H.)

**Keywords:** curcumin, breast cancer, cancer-associated fibroblast, tumor microenvironment, adipose-derived mesenchymal stem cell, CXCL12/CXCR4 axis

## Abstract

Adipose tissue has a significant impact on breast cancer initiation and progression owing to its substantial proportion in the breast. Adipose-derived mesenchymal stem cells (ADMSCs) are major players in the breast tumor microenvironment (TME) as they interact with cancer cells. The intricate interaction between ADMSCs and cancer cells not only drives the differentiation of ADMSCs into cancer-associated fibroblasts (CAFs) but also the metastasis of cancer cells, which is attributed to the CXCL12/CXCR4 axis. We investigated the effects of curcumin, a flavonoid known for CXCL12/CXCR4 axis inhibition, on breast TME by analyzing whether it can disrupt the ADMSC-cancer positive loop. Using MCF7 breast cancer cell-derived conditioned medium (MCF7-CM), we induced ADMSC transformation and verified that curcumin diminished the phenotypic change, inhibiting CAF marker expression. Additionally, curcumin suppressed the CXCL12/CXCR4 axis and its downstream signaling both in ADMSCs and MCF7 cells. The CM from ADMSCs, whose ADMSC-to-CAF transformation was repressed by the curcumin treatment, inhibited the positive feedback loop between ADMSCs and MCF7 as well as epithelial–mesenchymal transition in MCF7. Our study showed that curcumin is a potent anti-cancer agent that can remodel the breast TME, thereby restricting the ADMSC-cancer positive feedback loop associated with the CXCL12/CXCR4 axis.

## 1. Introduction

Breast cancer is the most common cancer type among women worldwide and causes a significant burden on public health. The breast tissue is composed of epithelial and adipose tissue, and malignancies primarily arise from the epithelial tissue via genetic mutations [1]. As the adipose tissue covers a predominant portion of breast tissue, the interaction between breast cancer cells and adipose tissue is inevitable when breast cancer occurs [2]. Among the constituents of adipose stromal tissue, adipose-derived mesenchymal stem cells (ADMSCs) exert a wide range of biological functions and play a major role in regulating the homeostasis of breast tissue. In normal breast tissue, they differentiate into diverse cell types, facilitating tissue repair and regeneration. However, when epithelial cells undergo genetic and epigenetic modification during tumorigenesis, ADMSCs are also influenced by the surrounding environment and result in phenotypic transition, including transformation into cancer-associated fibroblasts (CAFs) [3,4,5,6,7].

CAFs are characterized by their abnormal phenotype, including increased extracellular matrix component production, when compared to normal fibroblasts, and contribute to breast cancer progression [8]. Elevated cytokine and chemokine secretion by CAFs promotes a pro-tumorigenic microenvironment, which leads to cancer cell growth, survival, and invasion [9]. Among molecular pathways associated with the breast tumor microenvironment (TME), the C-X-C motif chemokine ligand 12 (CXCL12)/C-X-C chemokine receptor type 4 (CXCR4) axis emerges as a crucial signaling pathway because it induces breast cancer cell motility and is involved in most breast cancer metastases [10,11]. CXCL12 is a small chemokine protein secreted by various cells and tissues, including stromal cells, endothelial cells, and fibroblasts [12]. In breast cancer, CXCL12 is often overexpressed in the TME [13]. CXCR4 is a surface receptor protein that binds to CXCL12. It is expressed in many cell types, including immune cells, stem cells, and cancer cells [14]. CXCR4 activation triggers intracellular signaling pathways in the TME that are correlated with aggressive tumor behavior [15,16]. Owing to its role in cancer metastasis and progression, the CXCL12/CXCR4 axis is an important target for cancer therapy.

The nuclear factor kappa B (NF-κB) signaling pathway is downstream of CXCL12/CXCR4, modulating breast cancer progression along with inflammation, cell proliferation, migration, and invasion [17,18]. In addition, NF-κB activation induces the transcription of sonic hedgehog (SHH), a member of the hedgehog family of signaling proteins [19]. SHH is released as a soluble protein and acts as a morphogen, which helps to establish tissue patterns during development by influencing cell fate and behavior [20]. In cancer, a dysregulated NF-κB pathway leads to aberrant SHH expression [21]. Secreted SHH binds to protein patched homolog 1 (Ptch1), amplifying the expression and extracellular secretion of CXCL12 [22]. Therefore, the NF-κB-induced SHH upregulation is a crucial driver in initiating the positive feedback loop within the TME by affecting cellular behavior and tumor development. Considering the pivotal role of the CXCL12/CXCR4 axis in the complex TME, the development of targeted therapies aimed at disrupting this pathway is needed for efficient cancer treatment.

Curcumin is a natural polyphenolic compound derived from a turmeric plant (*Curcuma longa*). Its therapeutic effects against cancer have been well-documented, showing anti-cancer, anti-oxidative, and anti-inflammatory effects [23,24,25]. Moreover, previous studies have suggested that curcumin can interfere with the metastasis of cancer cells by inhibiting the CXCL12/CXCR4 axis [26,27]. Although the anti-cancer activity of curcumin is widely studied, it is necessary to investigate its capacity to reshape the breast TME by modulating the intricate network of downstream signaling pathways related to the CXCL12/CXCR4 axis.

This study aimed to identify the potential of curcumin as a therapeutic candidate for regulating breast TME via the CXCL12/CXCR4 axis. Curcumin effectively diminished the CXCL12/CXCR4 axis and its downstream signaling both in ADMSCs and MCF7 cells. Curcumin disrupted the positive feedback loop between ADMSCs and MCF7 cells and, in turn, disrupted the epithelial–mesenchymal transition (EMT) in MCF7 cells. Our findings suggest that curcumin has great value as an anticancer agent that can remodel breast TME by limiting ADMSC-cancer interaction through regulation of the CXCL12/CXCR4 axis.

## 2. Materials and Methods

### 2.1. Cell Culture and Collection of Conditioned Medium (CM)

MCF7 human breast cancer cells were purchased from the American Type Culture Collection (Manassas, VA, USA) and cultured in DMEM high glucose (Welgene, Gyeongsan, Republic of Korea) containing 10% FBS and 1% penicillin–streptomycin. Human ADMSCs were acquired from CEFO (Seoul, Republic of Korea) and cultured in ADMSC growth medium (CEFO). All cells were cultured in a humid incubator with 5% CO_2_ at 37 °C.

For the collection of CM from MCF7 (MCF7-CM), MCF7 cells were seeded in 100 mm^2^ cell culture dishes at a density of 2 × 10^6^ cells/dish. After 5 days of culture, MCF7-CM was collected from MCF7 and stored at −80 °C until use. For the following experiments, MCF7-CM was diluted with DMEM high glucose containing 10% FBS and 1% penicillin–streptomycin. To collect the CM from ADMSCs, cells were seeded in 100 mm^2^ cell culture dishes at a density of 2 × 10^6^ cells/dish and were cultured for 24 h. ADMSCs were treated with 20 μM AMD3100, dexamethasone, and curcumin (Sigma-Aldrich, St. Louis, MO, USA) in DMEM containing 50% MCF7-CM. After 72 h, cells were washed with PBS and incubated in DMEM high glucose (Gibco, Billings, MT, USA) containing 10% FBS and 1% penicillin–streptomycin. CM was harvested from the respective experimental groups, including the control, MCF7-CM-treated, AMD3100-treated, dexamethasone-treated, and curcumin-treated groups, which were designated as Con-CM, CAF-CM, AMD-CM, Dex-CM, and Cur-CM, respectively.

### 2.2. Cell Viability Assay

Cell viability assays were performed to evaluate the cytotoxicity of curcumin on ADMSCs using the Quanti-Max WST-8 Cell Viability Assay Solution (WST-8 Solution, Biomax, Seoul, Republic of Korea). ADMSCs were seeded in 96-well plates at a density of 10 × 10^4^ cells/mL. Various concentrations of curcumin with or without 50% MCF-CM for 72 h were used to treat the cells. The Quanti-Max WST-8 Cell Viability Assay Solution was added to each cell culture, followed by incubation of the plate for 1 h. Using a microplate reader (Molecular Devices, San Jose, CA, USA), the absorbance was measured at 450 nm, and the relative cell viability was determined.

### 2.3. mRNA Extraction and Quantitative Real-Time Polymerase Chain Reaction (qRT-PCR)

Total mRNA was isolated from cells using TRIzol (Life Technologies, Carlsbad, CA, USA), following the manufacturer’s instructions. The purity and concentration of isolated total mRNA were determined using the Nanodrop-2000 (Thermo Fisher Scientific, Waltham, MA, USA). The cDNA was synthesized using 2 μg of extracted total RNA and Reverse Transcription Master Premix (ELPIS, Daejeon, Republic of Korea). The qRT-PCR was performed using synthesized cDNA, primer sets, and SYBR Green PCR Master Mix (KAPA, Wilmington, MA, USA) via CFX Connect Real-Time PCR Detection System (Bio-Rad, Hercules, CA, USA).

### 2.4. Western Blotting Analysis

Total proteins were extracted using RIPA buffer (Bio Solution, Seoul, Republic of Korea) containing 2/3 phosphatase-inhibitor cocktails and protease inhibitor cocktail (Sigma-Aldrich). Protein quantification was carried out using the Pierce BCA Protein Assay Kit (Thermo Fisher Scientific). Equal amounts of protein from each sample were separated using 10% sodium dodecyl sulfate-polyacrylamide gel electrophoresis, and the separated proteins were then transferred to a polyvinylidene difluoride membrane (Millipore, Burlington, MA, USA).

The transferred membranes were blocked with 5% skim milk at room temperature for 1 h. Incubation with primary antibodies against vimentin, CXCL12, SHH, p-p65, GAPDH (Santa Cruz Biotechnology, Dallas, TX, USA), alpha-smooth muscle actin (α-SMA) (Cell Signaling Technology, Danvers, MA, USA), CXCR4 (GeneTex, Irvine, CA, USA), p65 (Abcam, Cambridge, UK), E-cadherin, N-cadherin (Proteintech, Rosemont, IL, USA), and fibronectin (Thermo Fisher Scientific) was performed overnight at 4 °C. The secondary antibodies conjugated with horseradish peroxidase were applied the next day at room temperature for 45 min after washing with 1 × TBST. Target proteins were detected using the ECL Plus Western blotting detection reagent (Amersham Bioscience, Buckinghamshire, UK), and images were captured using the Bio-Rad ChemiDoc XRS System (Hercules, CA, USA). To normalize the acquired data, GAPDH was used as a loading control, and data quantification was performed using Image Lab 4.1 Software (Bio-Rad, Hercules, CA, USA).

### 2.5. Immunocytochemistry Staining

After seeding on poly-L-lysine (Sigma-Aldrich)-coated cover slides, the cells were fixed with 4% formaldehyde (Sigma-Aldrich) for 10 min and were permeabilized with 0.25% Triton X-100 (Sigma-Aldrich) for 10 min. To block nonspecific binding sites, the samples were incubated with 2% bovine serum albumin (BSA, Santa Cruz Biotechnology) in PBS for 1 h at room temperature. The samples were incubated for 1 h at room temperature with either an anti-CXCR4 antibody, anti-p-p65 antibody, or anti-SHH antibody (1:500 in 1% BSA/TBST solution). After washing, anti-rabbit or anti-mouse Alexa Fluor 555 antibody (1:1000 in 1% BSA/TBST solution, Cell Signaling Technology) was added to the cells for 1 h at room temperature. Finally, the samples were stained with 1 μg/mL of 4′,6-diamidino-2-phenylindole (Sigma-Aldrich). The fluorescent images were observed using a confocal microscope (Carl Zeiss, Oberkochen, Germany), and quantification was performed using ImageJ software version 1.4.3 (National Institutes of Health, Bethesda, MD, USA).

### 2.6. Enzyme-Linked Immunosorbent Assay (ELISA)

To quantify SHH protein levels, the cell lysates were prepared from each experimental group. The cells were rinsed gently with pre-cooled PBS, followed by cell detachment using trypsin. The cell suspension was collected into a centrifuge tube and was centrifuged for 5 min at 1000× *g*. After washing three times, 1 × 10^6^ cells of each sample were resuspended with 250 μL of pre-cooled PBS. The freeze–thaw process was repeated three times until the cells were fully lysed. After centrifuging for 10 min at 1500× *g* at 4 °C, the supernatant was collected to carry out the assay. The SHH protein in the samples was quantified using the Human SHH (Sonic Hedgehog Protein) ELISA Kit (Elabscience, Houston, TX, USA), according to the manufacturer’s instructions.

### 2.7. Cell Migration and Invasion Assays

Cell migration assays were performed using Culture-insert 2 wells (Ibidi, Munich, Germany) to create equal gaps among the experimental groups. The inserts were first placed on 12-well plates, and 70 μL of MCF7 cells (1 × 10^6^ cells/mL) was then seeded into each insert well. The insert was carefully removed after cells reached 90% confluency, and Con-CM, CAF-CM, AMD-CM, and Cur-CM were added to each well. After 18 h, cell migration was observed using an inverted microscope (Leica Microsystems, Wetzlar, Germany), and the cell migration area was quantified using ImageJ software version 1.4.3.

Cell invasion assays were performed using a 96-well cell invasion assay kit (Cell Biolabs, San Diego, CA, USA). Cells were seeded onto the upper chambers of the 8 μm Transwell insert at a density of 2 × 10^5^ cells/well with serum-free media. The lower chamber was filled with a serum-containing medium to serve as an attractant, and the plate was incubated at 37 °C for 72 h. After incubation, the media in the upper chambers were aspirated. Invaded cells were detached using 1 × Cell Dissociation Solution and lysed with a lysis buffer. Cell lysates were stained with CyQuant GR dye solution, and fluorescence was measured with an Infinite F200 Pro multimode microplate reader (Tecan, Männedorf, Switzerland) at 485 nm excitation and 520 nm emission. The relative fluorescence signal was calculated in comparison to the controls to quantify the invasion properties of the samples.

### 2.8. Statistical Analysis

All experiments were performed in triplicate, and the results are presented as the mean ± standard error of the mean (SEM). To assess the statistical significance, a one-way ANOVA test followed by Tukey’s post-test was conducted using GraphPad Prism Software Version 5 (GraphPad Software, La Jolla, CA, USA). *p*-values < 0.05 were considered statistically significant.

## 3. Results

### 3.1. The Inhibitory Effect of Curcumin on the Transformation of ADMSCs into CAFs

**Figure 1:**
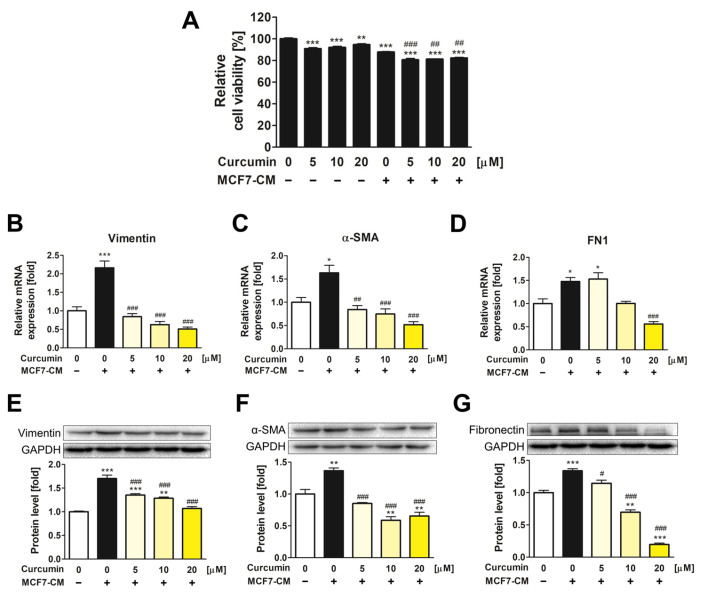
Curcumin inhibited the transformation of ADMSCs into CAFs. (**A**) Relative cell viability of curcumin and MCF7-CM on ADMSCs was measured using a WST-8 solution. ADMSCs were seeded, and various concentrations of curcumin were used to treat cells with or without 50% MCF-CM for 72 h. The gene expression of (**B**) vimentin, (**C**) α-SMA, and (**D**) FN1 was assessed by qRT-PCR. The protein levels of (**E**) vimentin, (**F**) α-SMA, and (**G**) fibronectin were measured via Western blotting analysis. * *p* < 0.05, ** *p* < 0.01, and *** *p* < 0.001 compared to the control group; # *p* < 0.05, ## *p* < 0.01, and ### *p* < 0.001 compared to the group treated only with MCF7-CM.

Before investigating the effects of curcumin on ADMSC-CAF transformation, cell viability assays were performed to evaluate the cytotoxicity of curcumin and MCF7-CM on ADMSCs (Figure 1A). ADMSCs were treated with various concentrations of curcumin, both with and without 50% MCF7-CM, for 72 h. Cell toxicity assays revealed no significant cytotoxic effects of curcumin or MCF7-CM on ADMSCs, with cell viability being above 80% in all experimental groups.

To validate whether MCF7-CM induces CAF transformation of ADMSCs, the mRNA and protein levels of CAF markers, including vimentin, α-SMA, and fibronectin (FN1), were determined (Figure 1B–G). The MCF7-CM treatment group without curcumin exhibited a significant upregulation of CAF markers, implying the successful induction of ADMSC transformation into CAFs by MCF7-CM. However, when ADMSCs were co-treated with curcumin and MCF7-CM, CAF-related genes were significantly downregulated compared with those in the MCF7-CM treatment group. The results showed that curcumin inhibited the phenotypic alteration of ADMSCs into CAFs.

### 3.2. The Downregulatory Impact of Curcumin on CXCL12 and CXCR4 Expression of ADMSCs during CAF Transformation

**Figure 2:**
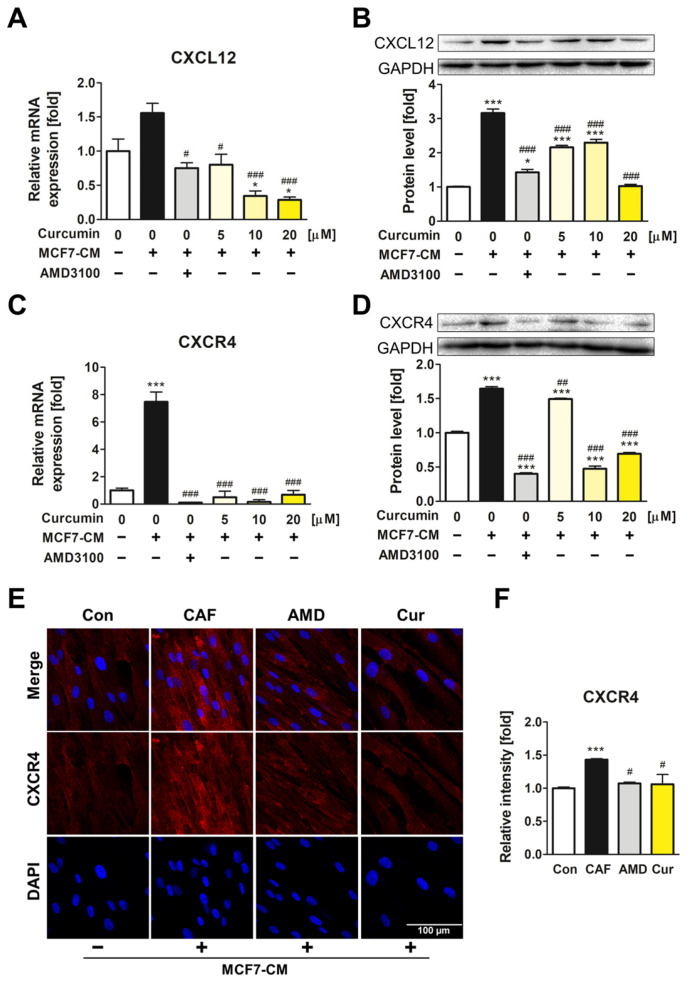
Curcumin downregulated CXCL12 and CXCR4 expression in MCF7-CM-treated ADMSCs. (**A**–**D**) The relative mRNA and protein levels of CXCL12 and CXCR4 were determined. Curcumin inhibited both mRNA and protein levels of CXCR4 and CXCL12. (**E**,**F**) The relative fluorescence intensity of CXCR4 on the cell surface of ADMSCs was determined and quantified. * *p* < 0.05 and *** *p* < 0.001 compared to the control group; # *p* < 0.05, ## *p* < 0.01, and ### *p* < 0.001 compared to the group treated only with MCF7-CM; Con: control; CAF: MCF7-CM-treated group; AMD: MCF7-CM-treated group with 20 μM of AMD3100; Cur: MCF7-CM-treated group with 20 μM of curcumin; scale bar: 100 μm.

The expression of CXCL12 and CXCR4 was determined to assess the potential of curcumin-mediated inhibition of the CXCL12/CXCR4 axis in ADMSCs (Figure 2A–D). In the MCF7-CM-treated group without curcumin, the expression of CXCL12 and CXCR4 was increased both in mRNA and protein levels. However, when cells were treated with curcumin and MCF7-CM, CXCR4 and CXCL12 expression was significantly downregulated compared to that of AMD3100 treatment, an antagonist of CXCR4. Immunocytochemistry results confirmed that CXCR4 expression on the ADMSC surface increased after MCF7-CM treatment, whereas its expression decreased after treatment with 20 μM of curcumin (Figure 2E), and this result was quantified (Figure 2F). These results implied that curcumin suppressed the conversion of ADMSCs into CAFs by effectively inhibiting the CXCL12/CXCR4 axis.

### 3.3. Repression of the NF-κB Signaling Pathway and SHH Expression by Curcumin during the ADMSC-CAF Transformation

**Figure 3:**
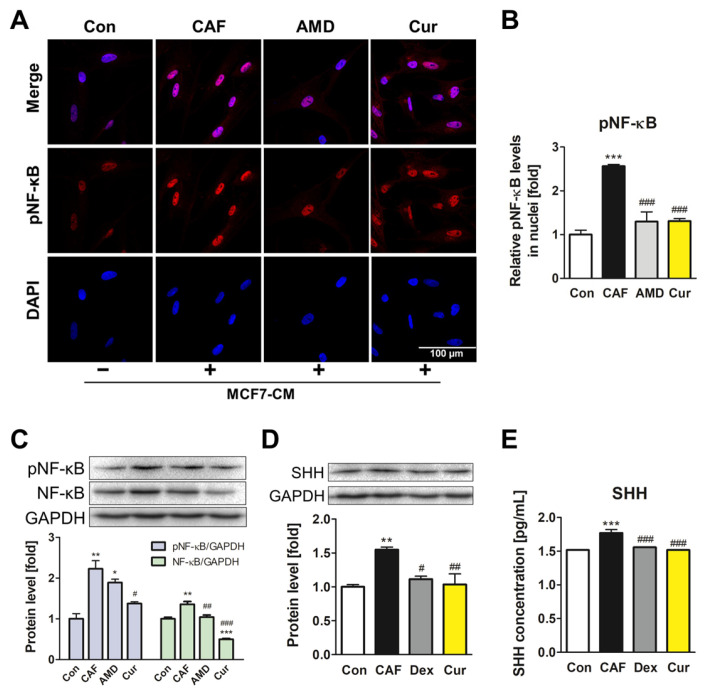
Curcumin inhibited the NF-κB signaling pathway and SHH expression, which are downstream of the CXCL12/CXCR4 axis in ADMSCs. (**A**,**B**) Relative pNF-κB translocation levels in nuclei of ADMSCs after treatment for 4 h were imaged via immunofluorescence staining and quantified. The protein levels of (**C**) pNF-κB, NF-κB, and (**D**) SHH were determined using Western blotting analysis. (**E**) The secretion level of SHH was quantified using ELISA kits. * *p* < 0.05, ** *p* < 0.01, and *** *p* < 0.001 compared to the control group; # *p* < 0.05, ## *p* < 0.01, and ### *p* < 0.001 compared to the CAF group; Con: control; CAF: MCF7-CM-treated group; AMD: MCF7-CM-treated group with 20 μM of AMD3100; Cur: MCF7-CM-treated group with 20 μM of curcumin; scale bar: 100 μm.

To investigate the effects of curcumin downstream of the CXCL12/CXCR4 axis, the expression and activity of NF-κB were determined. First, the effects of MCF7-CM were confirmed, as the treatment induced the activation of NF-κB in ADMSCs (Supplementary Figure S1). Among various time points (0–24 h), the 4 h treatment showed the highest upregulation of total NF-κB protein as well as phosphorylated NF-κB (pNF-κB) activation. Therefore, this time point was selected for further experiments regarding the NF-κB pathway. Immunofluorescence imaging showed that MCF7-CM increased the translocation of pNF-κB into nuclei, which was diminished by curcumin treatment, similar to the effect observed with AMD3100 treatment (Figure 3A,B). When curcumin was co-treated with MCF7-CM for 4 h, expression of both NF-κB and pNF-κB was suppressed by curcumin (Figure 3C). SHH expression is induced by NF-κB activation, which in turn promotes CXCL12 secretion in breast cancer cells and regulates the positive feedback loop in breast cancer TME. Indeed, in the present study, SHH protein expression was increased by MCF7-CM, whereas curcumin treatment reversed the effect. The curcumin-mediated downregulation of SHH was comparable to that of dexamethasone, a synthetic steroid with inhibitory activity against the NF-κB signaling pathway (Figure 3D). By measuring the secretion levels of SHH using ELISA, the increased SHH secretion induced by MCF7-CM decreased with curcumin and dexamethasone treatment, which is consistent with previous experiments (Figure 3E). The overall results indicated that curcumin modulated the secretion of SHH by inhibiting the activation of NF-κB signaling during ADMSC-CAF transformation.

### 3.4. Suppression of CXCL12/CXCR4 Axis and Its Downstream Effects in MCF7 by Curcumin

**Figure 4:**
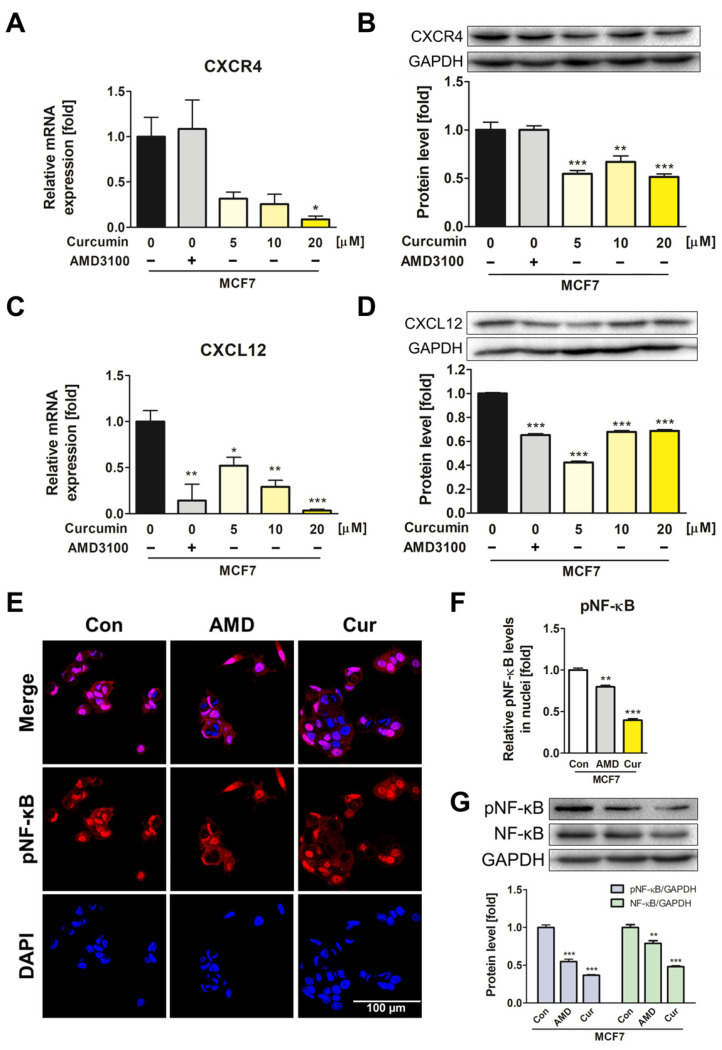
Curcumin suppressed the CXCL12/CXCR4 axis and its downstream elements in MCF7 cells. (**A**–**D**) The mRNA and protein levels of CXCL12 and CXCR4 were assessed after curcumin treatment at various concentrations for 72 h. (**E**,**F**) Immunocytochemistry analysis was performed to visualize and quantify the relative translocation levels of pNF-κB into the nuclei of MCF7 cells. (**G**) Western blotting analysis was conducted to assess the protein levels of pNF-κB and NF-κB after treatment for 4 h. * *p* < 0.05, ** *p* < 0.01, and *** *p* < 0.001 compared to the control group; Con: control; AMD: 20 μM of AMD3100-treated MCF7; Cur: 20 μM of curcumin-treated MCF7; scale bar: 100 μm.

As curcumin inhibited ADMSC transformation, we investigated whether curcumin also regulates breast cancer cell progression. MCF7 cells were treated with curcumin for 72 h, and the results showed significant downregulation of both mRNA and protein levels of CXCL12 and CXCR4 (Figure 4A–D). Notably, curcumin significantly downregulated the expression of CXCR4, whereas AMD3100 had a trivial effect.

In addition, the effects of curcumin on the NF-κB signaling pathway in MCF7 cells were investigated. Four-hour treatment of curcumin showed the most prominent inhibition of NF-κB expression and activity in MCF7 cells (Supplementary Figure S2). After curcumin treatment for 4 h, a significant decline in the translocation of pNF-κB into the cell nuclei was observed, resulting in a 0.5-fold reduction or lower compared with the control (Figure 4E,F). The expression levels of both pNF-κB and NF-κB were also significantly decreased by curcumin, which outperformed the inhibitory effect of AMD3100 treatment (Figure 4G). As curcumin exhibited an inhibitory effect on the CXCL12/CXCR4 axis and the NF-κB signaling pathway in MCF7 cells, it was demonstrated that curcumin has the potential to modulate breast cancer TME by repressing both ADMSCs and cancer cells.

### 3.5. Inhibition of CAF Transformation by Curcumin Suppresses the CXCL12/CXCR4 Axis-Mediated Positive Interaction between CAFs and MCF7 Cells

**Figure 5:**
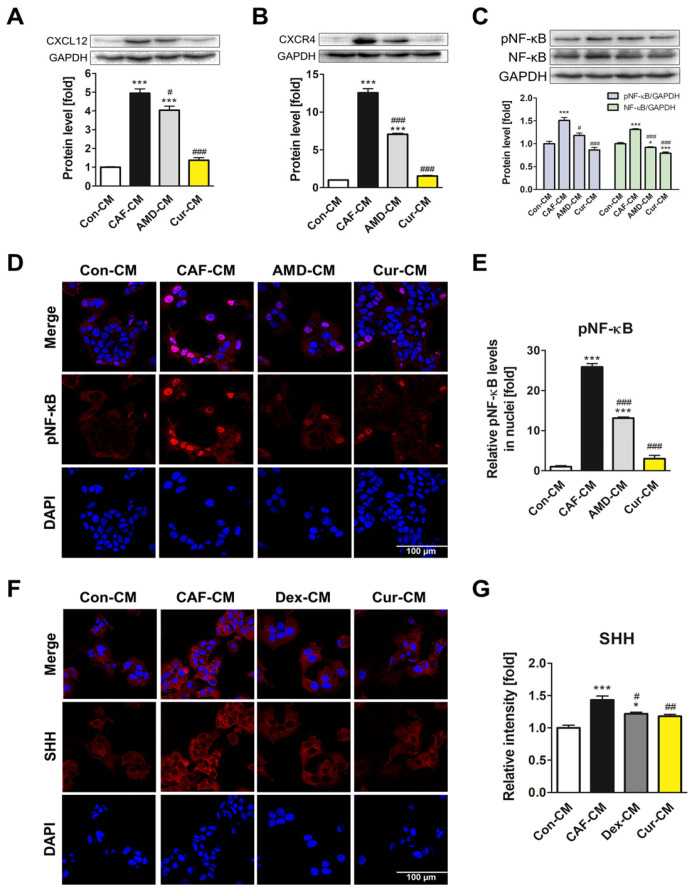
Curcumin-mediated inhibition of CAF transformation suppressed the positive interaction between CAFs and MCF7 cells mediated by the CXCL12/CXCR4 axis. The protein levels of (**A**) CXCL12 and (**B**) CXCR4 were evaluated following treatment with ADMSC-derived CM for 72 h. (**C**) Western blotting analysis was performed to examine the protein levels of pNF-κB and NF-κB following treatment with ADMSC-derived CM for 24 h. Immunofluorescence imaging was employed to visualize and quantitatively analyze (**D**,**E**) the relative translocation levels of pNF-κB in the nuclei and (**F**,**G**) SHH delivery levels on the cell surface of MCF7 cells following a 24 h treatment with ADMSC-derived CM. * *p* < 0.05 and *** *p* < 0.001 compared to the Con-CM-treated group; # *p* < 0.05, ## *p* < 0.01, and ### *p* < 0.001 compared to the CAF-CM-treated group; scale bar: 100 μm.

Next, we explored how inhibition of CXCL12/CXCR4 axis-mediated CAF transformation by curcumin affects MCF7 cells regarding the TME. After exposing ADMSCs to MCF7-CM for CAF transformation, CM from the transformed ADMSCs was collected and then used to treat MCF7 cells. Each CM was labeled as Con-CM, CAF-CM, AMD-CM, Dex-CM, and Cur-CM, based on which substance was added in MCF7-CM during the initial CM treatment. The Cur-CM treatment of MCF7 cells led to a decrease in CXCL12 and CXCR4 protein levels (Figure 5A,B). CAF-CM treatment for 24 h activated NF-κB signaling in MCF7 cells, with a 1.7-fold or higher upregulation of both pNF-κB and NF-κB (Supplementary Figure S3). When treated with CMs for 24 h, CAF-CM enhanced the expression and activation of NF-κB, whereas Cur-CM and AMD-CM significantly inhibited the NF-κB signaling pathway (Figure 5C). Confocal imaging showed a significant decrease in nuclear translocation of pNF-κB after treatment with Cur-CM, exceeding the inhibitory effect observed after AMD-CM treatment (Figure 5D,E). The fluorescent intensity of SHH was higher in CAF-CM-treated MCF7 cells but lower in Cur-CM- and Dex-CM-treated groups, indicating that the secretion of SHH was inhibited when ADMSCs were treated with curcumin and dexamethasone. The results showed that the delivery of SHH onto MCF7 cells was hindered as curcumin and dexamethasone suppressed NF-κB signaling (Figure 5F,G). It was suggested that curcumin disrupts the positive feedback loop mediated by CXCL12/CXCR4/NF-κB/SHH between CAFs and MCF7 cells, exhibiting its therapeutic potential by regulating the breast cancer TME.

### 3.6. Inhibiting CXCL12/CXCR4 Axis-Mediated Transformation of ADMSCs by Curcumin Reduced the Metastatic Potential of MCF7 Cells

**Figure 6:**
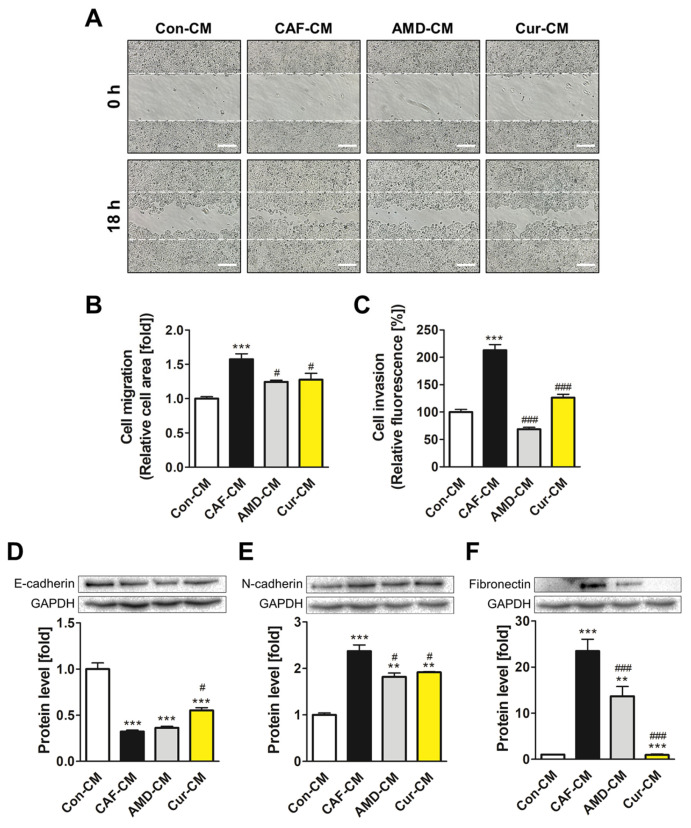
Curcumin-mediated inhibition of CAF transformation promoted by the CXCL12/CXCR4 axis hindered the EMT in MCF7 cells. (**A**,**B**) Cell migration assays were performed after creating equal gaps between the experimental groups and quantified. (**C**) The permeability of cells through the basement membrane was quantified using a fluorescence reader. The protein levels of (**D**) E-cadherin, (**E**) N-cadherin, and (**F**) fibronectin were determined using immunoblot assays. ** *p* < 0.01; and *** *p* < 0.001 compared to the Con-CM-treated group; # *p* < 0.05 and ### *p* < 0.001 compared to the CAF-CM-treated group; scale bar: 200 μm.

EMT plays a pivotal role in enhancing the mobility of cancer cells, facilitating their migration and invasion into surrounding tissues. We investigated whether the CXCL12/CXCR4 axis-mediated transformation of ADMSCs promotes the migration and invasion ability of MCF7 cells and whether curcumin reverses this tumor-promoting effect. The treatment with Cur-CM significantly reduced the migratory property of MCF7 cells when compared with the effects of Con-CM treatment (Figure 6A,B). Invasion assays revealed a substantial decrease in fluorescence signal from the Cur-CM treatment group, which showed a more than 50% reduction in cell invasion compared to that of the CAF-CM treatment group (Figure 6C). Furthermore, treatment with Cur-CM increased the expression of E-cadherin, an epithelial cell marker, while reducing the expression of N-cadherin and fibronectin, both mesenchymal cell markers (Figure 6D–F). The reversal of cell motility and EMT marker expression indicated that curcumin effectively interrupted the EMT induced by CAF-CM by inhibiting the mesenchymal-like features and promoting the epithelial characteristics of MCF7 cells.

## 4. Discussion

Breast cancer is a significant public health problem worldwide and is a leading cause of cancer occurrence and mortality in women. Treating breast cancer is difficult because of its complex TME and high recurrence and metastatic behavior [28]. Constituting stromal tissue within the normal breast, ADMSCs not only differentiate into various cell lineages but play a major role by secreting paracrine factors [29]. Under the influence of breast cancer, however, ADMSCs may transform into CAFs, affecting tumor development and metastasis [30]. Therefore, targeting CAFs is increasingly being considered in cancer research, as inhibiting their pro-tumorigenic properties impedes tumor progression and improves the efficacy of cancer treatment [31,32]. Notably, the CXCL12/CXCR4 axis holds significant value in cancer studies as it contributes to tumor development as well as the transition of ADMSCs into CAFs [33]. CXCL12, also referred to as stromal cell-derived factor-1 (SDF-1), enhances cancer cell metastasis via TGF-β1 secretion [34]. In addition, CXCR4 overexpression promotes cancer cell survival and metastasis, resulting in poor prognosis in patients with lung cancer [35]. As the CXCL12/CXCR4 axis mediates the interaction between stromal cells and cancer cells in TME, our study aimed to identify the therapeutic potential of curcumin by focusing on its inhibitory effect on breast TME.

Flavonoids are suitable candidates for cancer therapy because of their low toxicity and side effects compared with other anticancer drugs [36]. Moreover, they can be used in combination with conventional cancer treatments such as chemotherapy or radiation therapy [37]. Among various flavonoids, curcumin has been extensively studied in terms of cancer therapy owing to its diverse functions, which include inhibition of the CXCL12/CXCR4 axis, resulting in suppressed tumor metastasis [38]. Although the anticancer activities of curcumin have been well-documented, its applicability to breast TME is poorly understood. We first showed that such a suppressive effect on the CXCR4 pathway is independent of the anti-oxidative effect of curcumin (Supplementary Figure S4). ADMSCs were individually or co-treated with hydrogen peroxide (100 μM) and curcumin for 24 h. The results showed that oxidative stress induced by hydrogen peroxide neither affected the expression of CXCL12 and CXCR4 nor hindered the functionality of curcumin in ADMSCs. This implied that inhibition of the CXCL12/CXCR4 axis was not attributable to the antioxidant activity of curcumin but rather reflected a specific modulation of the axis. Therefore, we investigated the role of curcumin in ameliorating this complicated microenvironment of breast cancer by modulating the CXCL12/CXCR4 axis.

To establish an ADMSC-to-CAF transformation model, we utilized MCF7-CM, which was found to contain sufficient paracrine factors for driving such a transition. Upon MCF7-CM treatment, CAFs were characterized by upregulated expression of vimentin, α-SMA, and fibronectin, all of which are well-established markers of CAF. The significant downregulation of these CAF-associated genes was confirmed in curcumin-treated ADMSCs. Our results showed the ability of curcumin to modulate the transition of ADMSCs without adverse effects on cell viability. To observe that CAF transformation in the breast TME can be directed by the CXCL12/CXCR4 axis, the CXCR4 inhibitor AMD3100 was used to treat cells, and its effects were compared with those of curcumin treatment. The results showed that curcumin inhibited CAF transformation similarly to AMD3100, confirming the potential effect of curcumin on modulating the TME.

The NF-κB signaling pathway is downstream of the CXCL12/CXCR4 axis, and its activation contributes to cancer cell survival and invasion in various types of cancer, including prostate [39], pancreatic [40], lung [41], and breast cancer [42]. Research into NF-κB inhibitors has identified several promising natural compounds, including curcumin. The significant decrease in NF-κB expression observed in cells treated with curcumin indicated its impact on NF-κB in MCF7-CM-treated ADMSCs. This effect is associated with the anti-inflammatory and anti-cancer properties of curcumin [43]. In addition to its role in aggravating cancer, NF-κB activation induces a positive feedback loop within the TME through the upregulated expression of SHH [44]. Accordingly, a comprehensive understanding and targeting of CXCL12/CXCR4/NF-κB/SHH signaling holds great importance in treating cancer. Thereby, the suppressive role of curcumin on such a positive feedback loop was investigated. The activation of NF-κB signaling was initiated and sustained distinctively in different cell types and conditions. Accordingly, specific time points for observing the signaling activation were optimized in our study based on the expression of pNF-κB and NF-κB after CM treatment of ADMSCs or MCF7 cells. It was observed that SHH expression was effectively downregulated by curcumin during the transition of ADMSCs into CAFs by suppressing the expression and activation of NF-κB. To compare the effects of curcumin on SHH levels, dexamethasone, known as a direct inhibitor of NF-kB, was selected as a positive control. Curcumin treatment effects were comparable to those of the dexamethasone treatment in SHH expression and secretion, emphasizing that curcumin effectively interrupted the positive feedback loop in breast TME induced by NF-κB/SHH signaling.

Our research aimed not only to verify the impact of curcumin on CAF transformation but also to assess its effects on MCF7 cancer cells. Since MCF7 cells also overexpress CXCR4 and CXCL12 [45], the effects of curcumin on MCF7 cells in terms of the CXCL12/CXCR4 axis and its downstream elements were also investigated for the effective regulation of the breast TME. The treatment with curcumin reduced both mRNA and protein levels of CXCL12, which was similar to the AMD3100 treatment effects. Remarkably, although AMD3100 treatment did not affect CXCR4 expression, curcumin treatment induced a substantial reduction, demonstrating dual inhibitory effects on both the ligand and receptor. AMD3100 could not interfere with the production of CXCR4 but rather hindered its activation or internalization after it was expressed on the cell surface. In this case, the total expression levels would remain unchanged, while the availability of functional receptors could be diminished. Unlike AMD3100, curcumin can inhibit not only the activity of CXCR4 but also its expression in MCF7 cells. This suggested that curcumin showed a much greater ability to suppress the CXCL12/CXCR4 axis than the CXCR4 antagonist in MCF7 cells. In addition, we found that curcumin effectively inhibited NF-κB activation, which was most effective after 4 h of treatment. These results confirmed the role of curcumin in improving breast TME through its dual action on ADMSCs and cancer cells.

We subsequently explored the intricate interaction between ADMSCs and MCF7 breast cancer cells and the disruptive potential of curcumin in this interaction. Based on the established CAF transformation model using MCF7-CM with or without curcumin, CM from these ADMSCs was collected and used to treat MCF7 cells. The Cur-CM treatment effectively induced a significant reduction in CXCL12 and CXCR4 protein expressions in MCF7 cells. To gain deeper insights into the underlying molecular mechanisms, the temporal activation of the NF-κB signaling pathway in MCF7 cells induced by CAF-CM was determined. The results showed that the 24 h time point exhibited notable elevations in the expression of both pNF-κB and NF-κB. Cur-CM treatment displayed its regulatory effect by decreasing the protein levels of NF-κB and the translocation of pNF-κB into cell nuclei. SHH secreted by NF-κB activation subsequently binds to the Ptch1 receptor on MCF7 cells, which in turn increases the secretion of CXCL12. We observed that a significant amount of SHH was present in CAF-CM, and immunofluorescence assays confirmed the enhanced delivery of SHH onto the surface of MCF7 cells. In contrast, Cur-CM contained less SHH, where its treatment resulted in reduced delivery of SHH onto MCF7 cells when compared with CAF-CM. It was implied that curcumin inhibited the expression of SHH in ADMSCs by counteracting MCF7-CM, which ultimately led to the disruption of the NF-κB-SHH-mediated positive feedback cycle between CAFs and MCF7 cells.

The positive feedback loop in the breast TME contributes to the aggravation of cancer. Particularly, EMT facilitates the invasive and metastatic potential of tumors, which can be mediated by the NF-κB signaling pathway [46]. During EMT, cellular behavior undergoes notable shifts, with diminished expression of epithelial markers and increased mesenchymal-like phenotype [47]. To evaluate the impact of Cur-CM on EMT, the migratory and invasive capabilities of MCF7 cells were determined. Remarkably, treatment with Cur-CM significantly reduced the mobility and migration potential of MCF7 cells compared with Con-CM treatment. To further explore the molecular mechanism underlying such a phenotype, we examined the expression levels of EMT markers. E-cadherin, an epithelial cell marker, was upregulated following Cur-CM treatment. In contrast, the expression of N-cadherin and fibronectin, both indicators of mesenchymal characteristics, was significantly downregulated after Cur-CM treatment. These results explained that curcumin modulated the EMT by disrupting the CXCL12/CXCR4-mediated transformation of ADMSCs into CAFs and suppressing the breast TME from further progression.

Flavonoids possess substantial value when used in cancer therapy because of their broad functionalities, such as cell cycle regulation, anti-inflammatory effect, and apoptosis induction, that contribute to suppressing and preventing the disease. Another noteworthy benefit of flavonoids is their compatibility with existing anticancer medications [48]. Previous research reported that co-treatment of nanoparticle-based curcumin with conventional anticancer drugs increases intracellular drug levels and enhances the anticancer effect [49,50,51]. In addition to the known benefits of flavonoids, our study holds significant value in elucidating how flavonoids regulate the overall TME by affecting not only the cancer cell itself but also its interaction with the surrounding stroma. Through our research, it was demonstrated that curcumin is an effective modulator of breast TME. By disrupting the CXCL12/CXCR4 axis and the following NF-κB signaling pathway and EMT, curcumin alleviated the disease by mitigating the progression and metastasis of MCF7 and finally suppressing the positive loop formation within breast TME. These findings provide valuable insights into the therapeutic applications of curcumin and other flavonoids in treating cancer effectively in terms of tackling the TME.

## Data Availability

The data presented in this study are available in this article (and Supplementary Materials).

## Supplementary Materials

The following supporting information can be downloaded at: https://www.mdpi.com/article/10.3390/pharmaceutics15112627/s1, Table S1: The qRT-PCR primer sets utilized in this study; Figure S1: Assessment of the optimal time for NF-κB activation in ADMSCs when MCF7-CM was treated; Figure S2: Measurement of the optimum time for NF-κB inhibition in MCF7 cells after curcumin treatment; Figure S3: Determining the appropriate time for NF-κB activation in MCF7 cells following CAF-CM treatment; Figure S4: The inhibitory effect of curcumin on the CXCL12/CXCR4 axis is independent of its antioxidant properties.
