# Supplementary material for: Curcumin Disrupts a Positive Feedback Loop between ADMSCs and Cancer Cells in the Breast Tumor Microenvironment via the CXCL12/CXCR4 Axis
Source: Pharmaceutics. 2023 Nov 15;15(11):2627. doi: 10.3390/pharmaceutics15112627 (PMC10675183; doi:10.3390/pharmaceutics15112627)
Supplement: Supplementary file 1 [file pharmaceutics-15-02627-s001.zip › pharmaceutics-2694284-supplementary.pdf]

Supplementary Materials

Curcumin disrupts a positive feedback loop between ADMSCs and cancer cells in the breast tumor microenvironment via the CXCL12/CXCR4 axis

Table S1. The qRT-PCR primer sets utilized in this study.

Figure S1. Assessment of the optimal time for NF-κB activation in ADMSCs when MCF7-CM was treated.

Figure S2. Measurement of the optimum time for NF-κB inhibition in MCF7 cells after curcumin treatment.

Figure S3. Determining the appropriate time for NF-κB activation in MCF7 cells following CAF-CM treatment.

Figure S4. The inhibitory effect of curcumin on the CXCL12/CXCR4 axis is independent of its antioxidant properties.

Table S1. The qRT-PCR primer sets utilized in this study.

Gene	Sequence (5’ → 3’)	
**GAPDH**	F	TATGACAACAGCCTCAAGAT
	R	GAGTCCTTCCACGATACC
**Vimentin**	F	AGGCAAAGCAGGAGTCCACTGA
	R	ATCTGGCGTTCCAGGGACTCAT
**α-SMA**	F	GCTTGTCAGGGCTTGTCCA
	R	TTGAAGCGGAAGTCTGGGAA
**FN1**	F	ACAACACCGAGGTGACTGAGAC
	R	GGACACAACGATGCTTCCTGAG
**CXCL12**	F	TGCCCTTCAGATTGTAGCCC
	R	GCCCTTCCCTAACACTGGTT
**CXCR4**	F	CAGATAACTACACCGAGGAA
	R	TGACCAGGATGACCAATC

Figure S1. Assessment of the optimal time for NF-κB activation in ADMSCs when MCF7-CM was treated.

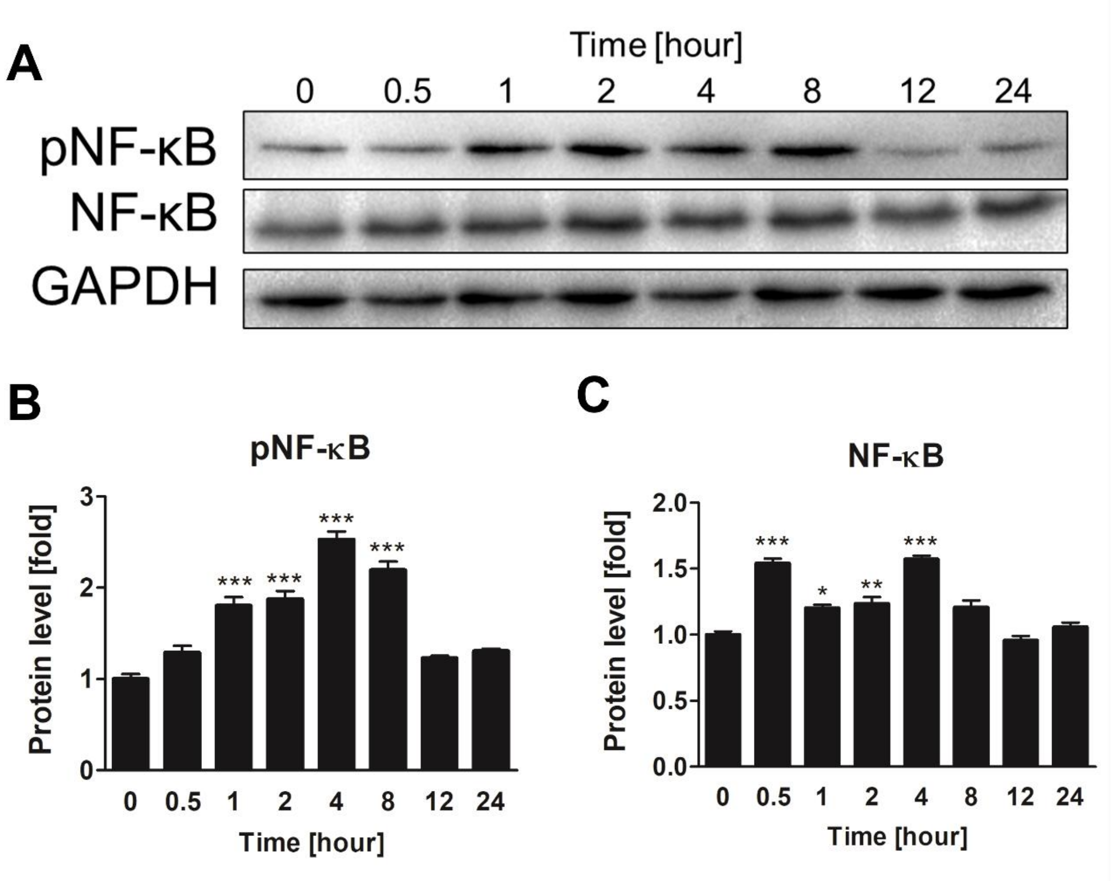

ADMSCs were exposed to 50% of MCF7-CM over a time range of 0-24 h to evaluate protein expressions of pNF-κB and NF-κB, which were subsequently quantified through immunoblot analysis. * p <0.05, ** p <0.01, and *** p <0.001 compared to the 0-hour group.

Figure S2. Measurement of the optimum time for NF-κB inhibition in MCF7 cells after curcumin treatment.

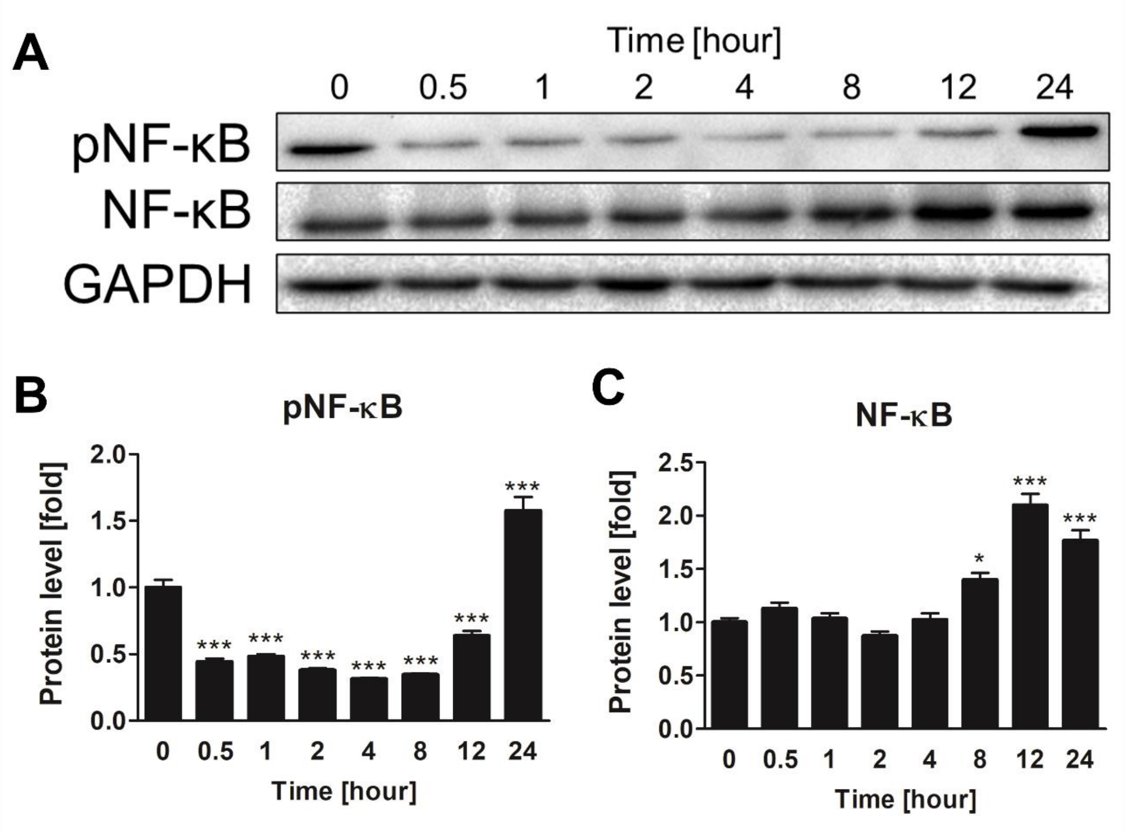

A concentration of 20 μM curcumin was treated to MCF7 breast cancer cells over a time ranging from 0 to 24 h. This treatment aimed to assess the protein expressions of pNF-κB and NF-κB.* p <0.05 and *** p <0.001 compared to the 0-hour group.

Figure S3. Determining the appropriate time for NF-κB activation in MCF7 cells following CAF-CM treatment.

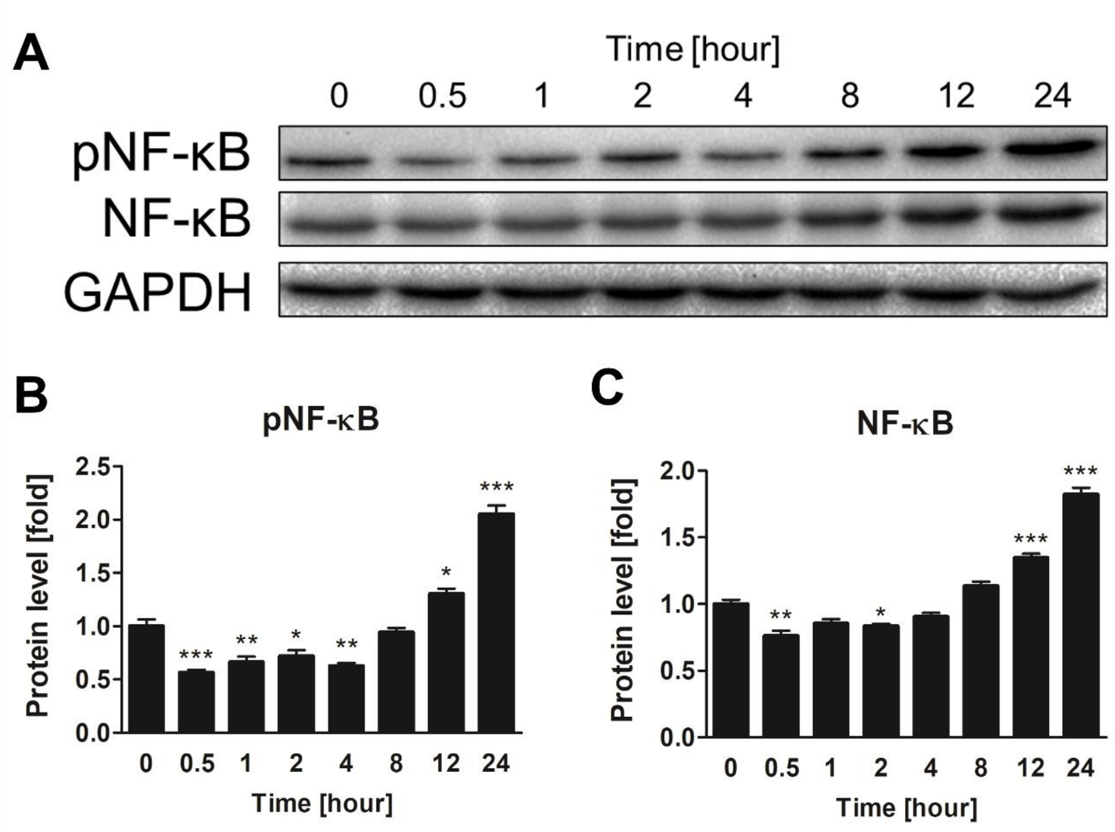

To determine the ideal time point for NF-κB activation in MCF7 cells following CAF-CM treatment, a time-course experiment was conducted. MCF7 cells were exposed to Cur-CM, and samples were collected at various time points ranging from 0 to 24 h. * p <0.05, ** p <0.01, and *** p <0.001 compared to the 0-hour group.

Figure S4. The inhibitory effect of curcumin on the CXCL12/CXCR4 axis is independent of its antioxidant properties.

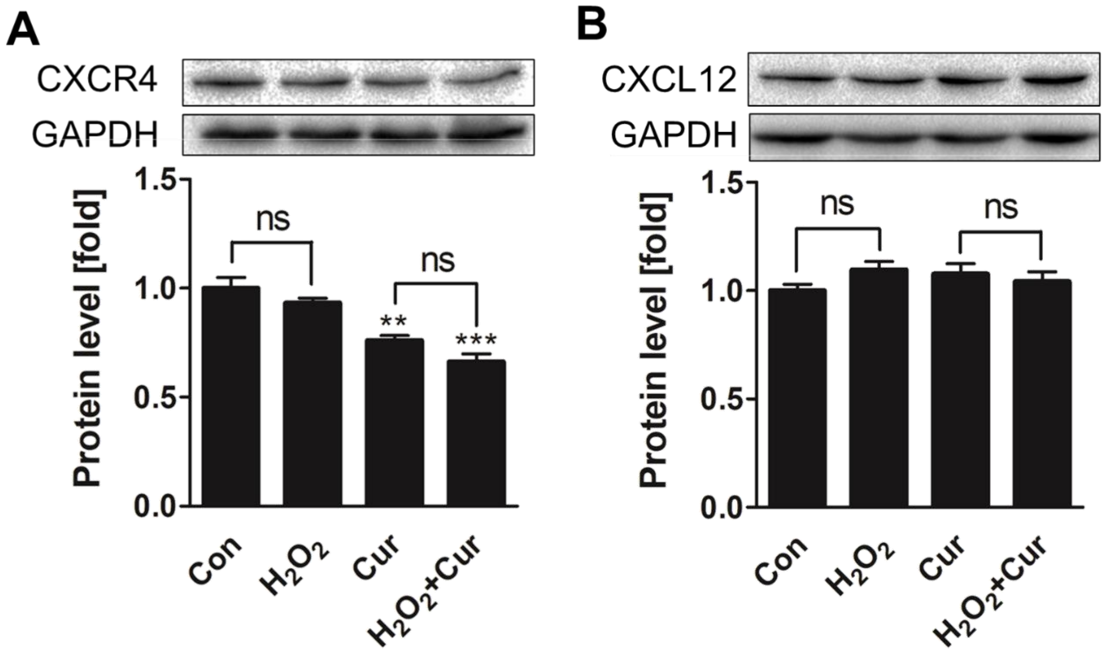

Protein levels of both (A) CXCR4 and (B) CXCL12 were assessed using an immunoblot assay. ADMSCs were seeded at a concentration of 1 x 10^5 cells/mL and subjected to treatment with 100 μM of hydrogen peroxide (H_2O_2) either alone or in combination with 20 μM of curcumin for 24 h. ** p <0.01 and *** p <0.001 compared to the control group; Con: control; H_2O_2: 100 μM of H_2O_2 treatment group; Cur: 20 μM of curcumin treatment group.
